# Additional K-ras mutation analysis and Plectin-1 staining improve the diagnostic accuracy of pancreatic solid mass in EUS-guided fine needle aspiration

**DOI:** 10.18632/oncotarget.16135

**Published:** 2017-03-11

**Authors:** Joo Kyung Park, Woo Hyun Paik, Byeong Jun Song, Ji Kon Ryu, Min A. Kim, Jin Myung Park, Sang Hyub Lee, Yong-Tae Kim

**Affiliations:** ^1^ Department of Medicine, Division of Gastroenterology, Samsung Medical Center, Sungkyunkwan University School of Medicine, Seoul, Korea; ^2^ Department of Internal Medicine, Inje University Ilsan Paik Hospital, Goyang, Korea; ^3^ Department of Internal Medicine, Myongji Hospital, Goyang, Korea; ^4^ Department of Internal Medicine, Seoul National University Hospital, Seoul National University College of Medicine, Seoul, Korea; ^5^ Department of Pathology, Seoul National University Hospital, Seoul National University College of Medicine, Seoul, Korea

**Keywords:** pancreatic ductal adenocarcinoma, endoscopic ultrasound, K-ras, Plectin-1

## Abstract

**Background:**

One of the major genetic alterations in pancreatic ductal adenocarcinoma (PDAC) is the point mutation of K-ras gene. Plectin-1 was also recently identified as PDAC specific biomarker. The aim of this study was to investigate the improvement of diagnostic accuracy of endoscopic ultrasound-guided fine needle aspiration (EUS-FNA) by using additional K-ras mutation analysis and Plectin-1 staining in patients with pancreatic mass.

**Methods:**

A total of 85 study patients with pancreatic mass underwent EUS-FNA and the final diagnoses were as follows; PDACs: 70 patients, pancreas neuroendocrine tumor: 4, metastasis to pancreas: 5, autoimmune pancreatitis: 3, chronic pancreatitis: 1, tuberculous lymphadenitis: 1, pseudocyst: 1.

**Results:**

Sensitivity, specificity and accuracy of pathologic diagnosis in EUS-FNA specimen were 81%, 80% and 79% accordingly. When we combine K-ras gene mutation analysis with histological assessment, we could get the following results for sensitivity, specificity and accuracy; cytology and K-ras mutation analysis: 93%, 87%, and 92%, cytology, K-ras mutation analysis, and Plectin-1 staining: 96%, 93%, and 95%.

**Conclusions:**

Triple combinations of the techniques; cytology, K-ras gene mutation analysis, Plectin-1 staining could increase accuracy in diagnosis of PDACs. Further investigation of using minimal specimens from EUS-FNA may give us insight to understand the biological behavior of PDAC.

## INTRODUCTION

Pancreatic cancer remains an incurable and rapidly lethal cancer, with a 5-year survival rate of less than 5% and a median survival of less than 1 year [1]. This grim prognosis is mostly due to the cancer's aggressive biological behavior with early invasion and metastasis, leading to an initial diagnosis at an advanced incurable stage in more than 80% of patients [2]. After high throughput next-generation sequencing technique has been introduced, the genetic evolution of pancreatic ductal adenocarcinoma (PDAC) has revealed that it takes 15 to 20 years to develop, even those patients undergoing potentially curative pancreatic resection will die of metastatic disease [3]. Despite recent discovery, still little is known about biology of PDAC progression due to the problems with tissue availability and usually insufficient quantity of tissue available for diagnosis and study. Also, several uncommon primary pancreatic tumors (pancreatic neuroendocrine tumors, pancreatic cystic neoplasms), inflammatory conditions (focal chronic pancreatitis, autoimmune and groove pancreatitis), metastasis to the pancreas and peripancreatic masses can mimic the appearance of PDAC [4]. Endoscopic ultrasound-guided fine-needle aspiration (EUS-FNA) is the most helpful tool for differentiation between these lesions and PDAC while avoiding unnecessary surgery. Therefore, EUS-FNA has become one of the most important diagnostic modality to confirm tissue diagnosis in patients with pancreatic mass. The diagnostic yield of EUS-FNA for solid pancreatic masses is 70% to 83% [5–9]. To improve diagnostic accuracy and acquire sufficient quantity of tissue from EUS-FNA are the corner stone of the evaluation and treatment of the patient with suspected pancreatic cancer. During pancreatic cancer tumorigenesis, many genetic and epigenetic alterations occur. One of the key features of genetic basis of PDAC is the point mutation of KRAS oncogene occurred in over 90 % of its pathogenesis [10]. **Many** studies have reported that *KRAS* mutation analysis with EUS-FNA appears to be very accurate at differentiating between benign and malignant pancreatic lesions [11–17]. Plectin-1 was also recently identified as PDAC specific biomarker [18]. It was reported that Plectin-1 was helpful to identify primary and metastatic PDAC and detect preinvasive pancreatic intraepithelial lesions-3 (PanIN III)[19].

The aim of presented work was to investigate techniques using minimal specimen acquired by EUS-FNA in order to achieve accurate diagnosis in patients with suspected PDACs. The key of this study was on finding an ideal combination in optimum results when used in combination with commonly used pathologic readings.

## RESULTS

### Baseline characteristics of study patients

Clinical information and laboratory data was reviewed among the 85 study patients (Table 1). The final diagnoses were as follows; PDACs: 70 patients, pancreas neuroendocrine tumor (PNET): 4, metastasis to pancreas: 5, autoimmune pancreatitis: 3, chronic pancreatitis: 1, tuberculous lymphadenitis: 1, pseudocyst: 1. The median age was 66 years old and male to female ratio was 38 to 47. There were 52 (61%) patients who had elevation of CA 19-9 serum level and their median level was 80 IU/ml ranged from 5 to 19,210 IU/ml. Also, 16 (19%) patients had elevated bilirubin and the median level was 0.75 mg/dL ranged from 0.3 mg/dL to 15.1 mg/dL. The location of pancreatic mass was as follows; head 40 patients (47%), body 25 (29%) and tail 20 (24%). Twenty-four patients (28%) had diabetes mellitus at the time of diagnosis of pancreatic mass and 15 patients had diabetes mellitus at least 3 years.

**Table 1:** Baseline characteristics of study patients

Age	Median	66 years
Sex	M:F	38:47
CA 19-9 (IU/mL)	Median (range)	80 (5∼19,210)
	elevated patients	52 (61%)
Bilirubin, Total (mg/dL)	median (range)	0.75 (0.3∼15.1)
	elevated patients	16 (19%)
Location (pancreas mass)	Head	40 (47%)
	Body	25 (29%)
	Tail	20 (24%)
DM (number of patients)	Number	24 (28%)
DM (onset time)	< 1 year	4
	1 ∼ 2 years	5
	≥ 3 years	15
Smoking	Number (median PYR)	11 (30 PYR)
Alcohol drinking	Heavy drinker*	14 (2%)
Family history of pancreatic cancer	1st degree	2

*Daily alcohol consumption more than 50 g.

### Characteristics of patients with EUS-FNA specimens

Characteristics of the EUS-FNA specimens are depicted in Table 2. EUS-FNA was conducted on all 85 patients without any complication (71 patients with a 22-gauge needle, 14 patients with a 25-gauge needle). A mean of 243 ng of DNA (range, 60-34590 ng) was obtained, and a mean *DNA concentration* was 81 ng/uL (range, 2-1183 ng/uL).

**Table 2:** Characteristics of patients with endoscopic ultrasound-guided fine needle aspiration (EUS-FNA) specimens

Complications	Number of cases	0
FNA needle	22-gauge	71
	25-guage	14
DNA extraction	concentration	81 (2∼1183 ng/uL)
	Total amount	243 ng (60∼35,490 ng)
Patients with pancreatic ductal adenocarcinoma	Number (%)	70 (82%)

### KRAS mutation analysis and Plectin-1 immunostaining of EUS-FNA specimens

Mutation analyses for KRAS (codon12/13) were successful in the 60 cases, and 25 cases were excluded from KRAS mutation analysis due to inadequate specimen. Thirty-nine patients showed a specific mutation of KRAS codon12 with a single base change from GGT (Gly) to GAT (Asp). 22 patients showed change from GGT (Gly) to GTT (Val), 4 patients to CGT (Arg), and 9 patients to GCT(Ala). No mutation could be detected in other tissues except 1 ampulla of Vater adenoma high grade and 1 pancreatic neuroendocrine carcinoma (Table 3). By adding KRAS mutation analysis, the diagnosis changed correctly in nine cases: eight patients with PDAC and one patient with chronic pancreatitis.

**Table 3:** *KRAS* mutation analysis of endoscopic ultrasound-guided fine needle aspiration specimens

Codon	Point mutation		Number of patients (%)
Codon 12	GGT (Gly)	GAT (Asp)	39
		GTT (Val)	22 (4)+
		CGT (Arg)	4 (3)+
		AGT (Ser)	0
		TGT (Cys)	0
		GCT (Ala)	9 (7)+
Codon 13	GGC (Gly)	GAC (Asp)	0
			60 Total*

*KRAS mutations: 58: pancreatic ductal adenocarcinomas, 2: other malignancies (Ampulla of Vater adenoma high grade and pancreatic neuroendocrine carcinoma).

+ Numbers in parenthesis indicate patients with more than 1 *KRAS* mutations.

**Figure 1:**
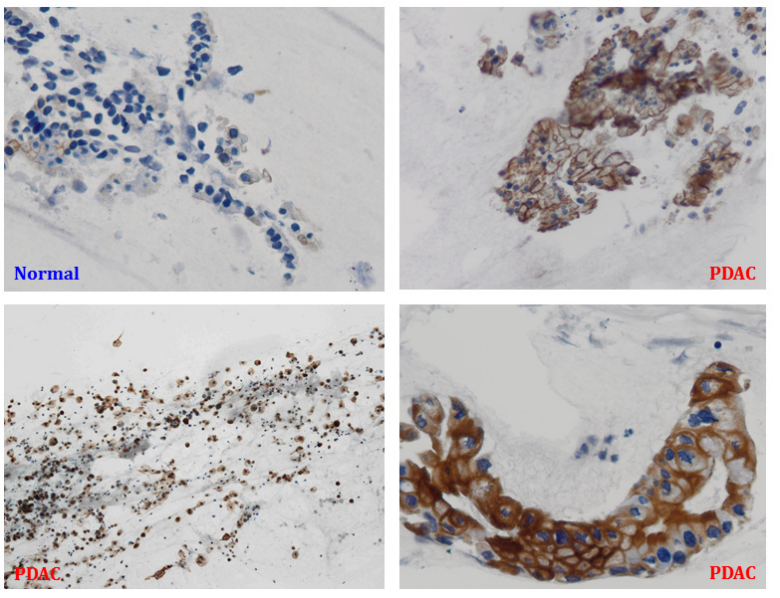
Plectin-1 immunostaining of endoscopic ultrasound-guided fine needle aspiration specimens PDAC, pancreatic ductal adenocarcinoma.

Normal pancreas did not express Plectin-1. Plectin-1 was identified in 100% (70/70) of PDACs (Figure 1). Only one case of PNET showed false positive with Plectin-1 immunostaining. By adding Plectin-1 immunostaining, the diagnosis changed correctly in two cases with PDACs..

### EUS-FNA cytology combined with other analyses

There were thirteen cases of PDACs with cytology dictating “atypical cells” or “inadequate for diagnosis. Among them, there were eight patients who had positive for K-ras mutation analysis and all 13 cases with PDACs were positive for Plectin-1 staining. On the other hand, there was no positive KRAS mutation detected in other cases rather than PDACs, but one case with PNET had positive for Plectin-1 staining. Since the cytopathologic examination was the gold standard for the final diagnosis, we made the final diagnosis based on cytopathologic result. As long as the cytopathologic results were definite and sufficient for evaluation, we did not consider the result of KRAS mutation analysis or Plectin-1 staining alone. Therefore, the sensitivity, specificity and accuracy of pathologic diagnosis in EUS-FNA specimens were 81%, 80% and 81%. When we combine KRAS mutation analysis with histological assessment, we could get the following results for sensitivity, specificity and accuracy; cytology & KRAS mutation analysis: 93%, 87%, and 92%, cytology, KRAS mutation analysis & Plec1 staining: 96%, 93%, and 95% (*p* < 0.05) (Table 4).

**Table 4:** Endoscopic ultrasound-guided fine needle aspiration cytology combined with other analyses

	Cytology	Cytology & KRAS mutation	Cytology, KRAS mutation & Plectin-1 staining	*p* value
Sensitivity	81%	93%	96%	0.003
Specificity	80%	87%	93%	0.002
Accuracy	79%	92%	95%	0.002

### Overall survival of PDAC patients according to KRAS mutation status

**Figure 2:**
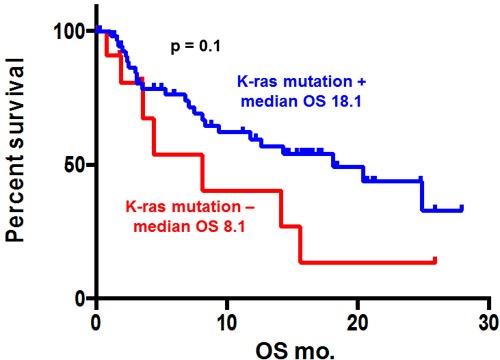
Kaplan-Meier overall survival (OS) curve of patients with pancreatic ductal adenocarcinoma according to *KRAS* mutation status KRAS mutation was not significantly associated with median overall survival (18.1 vs. 8.1 months, *p* = 0.1).

**Figure 3:**
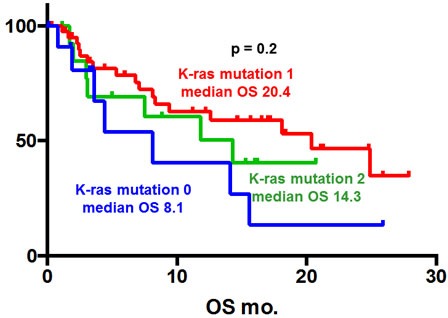
Kaplan-Meier overall survival (OS) curve of patients with pancreatic ductal adenomcarcinoma according to number of *KRAS* mutation There was no statistically significant differences about number of mutation in median OS (wild-type KRAS *vs* 1 KRAS mutation *vs* 2 KRAS mutation: Figure 3, 8.1 *vs* 20.4 *vs* 14.3 months, *p =* 0.2).

Of the 70 patients who were analyzed, there was no statistically significant differences in median overall survival (OS) (patients with KRAS mutations *vs* those with wild-type KRAS. Figure 2, 18.1 *vs* 8.1 months, *p =* 0.1). Also, there was no statistically significant differences about number of mutation in median OS (wild-type KRAS *vs* 1 KRAS mutation *vs* 2 KRAS mutation: Figure 3, 8.1 *vs* 20.4 *vs* 14.3 months, *p =* 0.2).

## DISCUSSION

Despite the recent advances in radiology, about 50% of all PDAC patients are found to have metastatic disease, surgical resection can be performed in only about 20%.[20] So, curative resection is dependent on early diagnosis. EUS-FNA is a very important method for diagnosis of the early pancreatic cancer. In order to improve the diagnostic accuracy of EUS-FNA of pancreatic masses, the most studied molecular tool is KRAS. Previous studies [11–17] used *KRAS* mutation analysis with EUS-FNA. Additionally we combined new biomarker Plectin-1. In this study, we examined the usefulness of combinations of conventional cytology, KRAS mutation analysis, and Plectin-1 staining for the diagnosis of solid pancreatic masses. Combinations of the techniques could increase sensitivity, specificity and accuracy in diagnosis of PDACs (96%, 93%, and 95%) as compared with conventional cytology (81%, 80% and 79%). More accurate diagnosis for pancreatic masses was able with KRAS mutation analysis and Plectin-1 staining. So, we established new helpful platform with EUS-FNA for diagnosis of PDACs. Plectin-1 is known to distinguish malignant intraductal papillary mucinous neoplasm (IPMN) from the benign IPMN, and also distinguish from PanIN I/II to PanIN III and PDACs.[19, 21] Further study about the diagnostic role of Plectin-1 in differentiating malignant IPMN, PanIN III, and PDAC with EUS-FNA samples is warranted.

Considering low negative predictive value of EUS-FNA in pancreatic malignancy and possibility of peritoneal seeding, some doctors resected pancreatic masses without EUS-FNA.[22] But, there are many benign lesions and various tumors in pancreas. Furthermore, a study showed that preoperative EUS-FNA in resectable pancreatic cancer was a safe tool.[23] In contrast, in patients with clinical suspicion of malignancy, a negative EUS-FNA cytology result does not provide conclusion for benign disease. So, clinicians should decide following closely or resecting pancreatic masses. Our study can reduce false-negative diagnoses and help to avoid the loss of surgical respectability. But, our study also has false negative data. So, in patients with high clinical suspicion of malignancy, surgeons might go on surgery regardless of EUS-FNA report. Some studies showed cyst fluid analysis with KRAS mutation also seems to be a good diagnostic method to determine the malignant potential of pancreatic cystic lesions. The accuracy in distinguishing malignant from benign cysts remains inadequate.[24] A study showed specificity of 96% for malignancy by KRAS mutation analysis and demonstrated that nonmalignant cysts by conventional cytology could be diagnosed as malignant.[25] Due to the progress of molecular biology, many studies about markers related with development of PDAC have been reported. EUS-FNA cytology combined with evaluation of KRAS mutations and allelic losses of tumor suppressor p16 and DPC4 is a very sensitive method in inconclusive cases.[26] MicroRNAs (miR) are promising molecular markers in PDAC that can be acquired with EUS-FNA. MiR-10b and miR-21 are over expressed in the FNA specimens from pancreatic cancer patients.[27] Also, the aspect of mucine (MUC) expression has a value for the diagnosis of PDAC. The panel MUC1+/ MUC5AC+ in EUS-FNA specimens is higher specific in the diagnosis of PDAC.[28] But, clinical use of miRNA and MUC is still debated. By extension, molecular marker acquired with EUS-FNA can influence the prognosis of PDAC and predict the response to therapy. Although there was no statistically significant differences in survival related to KRAS mutation status in our study (patients with KRAS mutations *vs* those with wild-type KRAS: 18.1 *vs* 8.1 months, *p =* 0.1), some studies demonstrated that the presence of KRAS mutations in tumor have a significant worse impact on survival time and response of treatment.[29] It is still difficult to conclude that the presence of KRAS mutations relates with the prognosis of advanced PDAC. To gain definitive conclusion, more studies containing large cohort are needed.

## MATERIALS AND METHODS

### Patients

**Figure 4:**
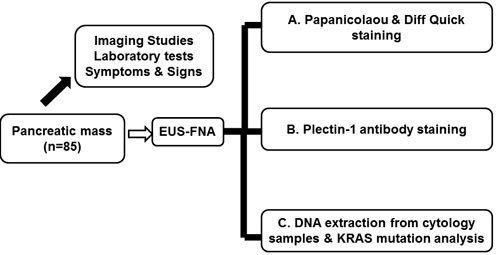
Schematic flow chart of study design

A total of 85 patients with pancreatic mass were consecutively enrolled in this study between June 2011 and July 2012 (Fig 4). The inclusion criteria were as follows: 1) age older than 19 years; 2) identifiable pancreatic solid mass by computed tomography (CT) scan; and 3) patients with informed consent. The exclusion criteria were as follows: 1) a cystic lesion; 2) patients with bleeding tendency (platelet count < 50,000/mm^3^ and/or prothrombin time international normalized ratio > 1.5); 3) resectable pancreatic masses highly suspected of PDACs in radiological imaging; and 4) refusal to participate in this study. Informed consents were obtained before the procedure. Demographic and clinical parameters were acquired from the electronic medical record, and the study protocol was approved by Institution Review Board of Seoul National University Hospital, Seoul National University College of Medicine (IRB NO: H-1202-070-398). All procedures and data acquisition were performed in accordance with STROBE statement.

### EUS-FNA procedure and acquisition of the specimens

EUS-FNA specimen was acquired by two experienced endoscopists (Professors: J.K.R. and Y.-T.K.) under the conscious sedation using meperidine and midazolam in pancreatobiliary clinic of GI division at Seoul National University Hospital. After the sedation, pancreatic mass was identified and explored the optimal site for EUS-FNA. The procedure was carried out using a linear array echo endoscope (GF-UM-2000 or GF-UCT 240; Olympus, Tokyo, Japan) with the 22G FNA needle (EchoTip Ultra; Wilson Cook Medical Inc.). EUS-FNA was performed as follows; after proper targeting to a mass, the FNA needle was passed through the biopsy channel and advanced into the target lesion under EUS guidance. To-and-fro movements were then performed 10 to 20 times with suction using a 10-mL syringe. After retracting the needle, the aspirated contents were expressed onto glass slides with the stylet smeared, and fixed in 99% ethanol. Pancreas mass located head or uncinate process was acquired by transduodenal passes while the body or tail mass was acquired through the puncturing stomach wall. The endoscopists assessed the gross adequacy of the sample, and selected the number of needle passes due to the absence of on-site cytopathologist. Additional needle passes to obtain study samples were not needed.

### Cytologic evaluations and immunostaining for Plectin-1

The aspirated specimen was expelled onto a glass slide by reinsertion of the stylet and flushing with air, if needed. Ten to fifteen slides were stained with alcohol and prepared using Papanicolaou & Diff Quick cytological stain to describe the cellularity and to diagnose malignancy in each specimen. There were three experts of pathology who immediately evaluated the specimens. Slides were evaluated using a Y-FL microscope (Nikon, Japan). The pathology reports of EUS-FNA are as follows: (1) positive for malignant cells (2) suspicious for malignant cells (3) atypical cells (4) benign cells (5) inadequate for diagnosis. Hence, the cytological results were interpreted as follows: (i) if the results were positive for malignant cells or suspicious for malignant cells, the masses were considered as malignant; (ii) if the results were atypical cells or inadequate for diagnosis, the masses were considered as malignant when other pathologic diagnosis with surgery, ERCP, or sono-guided/CT-guided biopsy was positive for malignant or clinical and imaging follow-up was consistent with malignancy, such as clinical progression or metastasis; (iii) if the results were atypical cells, benign cells, or inadequate for diagnosis, the masses were considered as benign based on the clinical manifestation (spontaneous improvement or lack of progression on a clinical minimum of six months follow-up). Plectin-1 (Cell Signaling, Danvers, MA, USA) antibody was used for IHC analyses. To determine intensity of Plectin-1 expression, nerve tissue stained with Plectin-1 was used as a positive control and reference. Nerves were known to have a moderate staining intensity. Staining intensity was recorded by two independent pathologists without giving clinical information and results from Papanicolaou & Diff Quick cytological stain. If there was any case of discrepant result, it was evaluated by a third pathologist. Plectin-1 staining was classified as negative if the staining intensity was weaker than nerves. It was classified as positive if the staining was as least as strong as nerves.

### KRAS mutation analysis of EUS FNA specimens

The DNA was recovered from FNA cytology slide to detect KRAS mutation. Mutant KRAS was detected using a validated KRAS mutation kit (DxS Ltd, Manchester, United Kingdom) that identifies seven somatic mutations located in codons 12 and 13 (Gly12Asp, Gly12Ala, Gly12Val, Gly12Ser, Gly12Arg, Gly12Cys, and Gly13Asp) using allele-specific real-time polymerase chain reaction [30–32]. A central laboratory (HistoGeneX, Antwerp, Belgium) validated the assay for analytic and diagnostic performance, established acceptance criteria, included appropriate quality controls for each assay, and performed the KRAS analysis in a blinded fashion.

### Statistical analyses

Continuous variables were described by mean and standard deviation (normal distribution), and the Wilcoxon rank sum test was done if normality could not be demonstrated. Differences across groups were compared using Student's t or the Mann-Whitney U test for continuous variables and the Fisher's exact test for binary variables. Correlation between continuous variables was assessed using linear regression models. All tests were two-tailed; in order to minimize overall type I error, only select associations were tested for statistical significance. Concerning the diagnosis obtained by EUS-FNA, sensitivity, specificity, and accuracy were calculated. Overall survival was analyzed by the Kaplan-Meier method, with use of one-sided log-rank statistics. The *p* values < 0.05 was considered statistically significant. All statistical analysis was performed using SPSS v.18.0 (IBM Corp., Armonk, NY, USA).

## References

[1] Jemal A, Siegel R, Ward E, Murray T, Xu J, Thun MJ (2007). Cancer statistics, 2007. CA Cancer J Clin.

[2] Koorstra JB, Hustinx SR, Offerhaus GJ, Maitra A (2008). Pancreatic carcinogenesis. Pancreatology.

[3] Yachida S, Jones S, Bozic I, Antal T, Leary R, Fu B, Kamiyama M, Hruban RH, Eshleman JR, Nowak MA, Velculescu VE, Kinzler KW, Vogelstein B (2010). Distant metastasis occurs late during the genetic evolution of pancreatic cancer. Nature.

[4] Buscail L, Faure P, Bournet B, Selves J, Escourrou J (2006). Interventional endoscopic ultrasound in pancreatic diseases. Pancreatology.

[5] Fisher L, Segarajasingam DS, Stewart C, Deboer WB, Yusoff IF (2009). Endoscopic ultrasound guided fine needle aspiration of solid pancreatic lesions: Performance and outcomes. J Gastroenterol Hepatol.

[6] Ramirez-Luna MA, Zepeda-Gomez S, Chavez-Tapia NC, Tellez-Avila FI (2008). Diagnostic yield and therapeutic impact of fine-needle aspiration biopsies guided by endoscopic ultrasound in pancreatic lesions. Rev Invest Clin.

[7] Savides TJ, Donohue M, Hunt G, Al-Haddad M, Aslanian H, Ben-Menachem T, Chen VK, Coyle W, Deutsch J, DeWitt J, Dhawan M, Eckardt A, Eloubeidi M (2007). EUS-guided FNA diagnostic yield of malignancy in solid pancreatic masses: a benchmark for quality performance measurement. Gastrointest Endosc.

[8] Turner BG, Cizginer S, Agarwal D, Yang J, Pitman MB, Brugge WR (2010). Diagnosis of pancreatic neoplasia with EUS and FNA: a report of accuracy. Gastrointest Endosc.

[9] Kida M, Araki M, Miyazawa S, Ikeda H, Kikuchi H, Watanabe M, Imaizumi H, Koizumi W (2011). Fine needle aspiration using forward-viewing endoscopic ultrasonography. Endoscopy.

[10] Almoguera C, Shibata D, Forrester K, Martin J, Arnheim N, Perucho M (1988). Most human carcinomas of the exocrine pancreas contain mutant c-K-ras genes. Cell.

[11] Bournet B, Souque A, Senesse P, Assenat E, Barthet M, Lesavre N, Aubert A, O'Toole D, Hammel P, Levy P, Ruszniewski P, Bouisson M, Escourrou J (2009). Endoscopic ultrasound-guided fine-needle aspiration biopsy coupled with KRAS mutation assay to distinguish pancreatic cancer from pseudotumoral chronic pancreatitis. Endoscopy.

[12] Ogura T, Yamao K, Sawaki A, Mizuno N, Hara K, Hijioka S, Niwa Y, Tajika M, Kondo S, Shimizu Y, Bhatia V, Higuchi K, Hosoda W (2012). Clinical impact of K-ras mutation analysis in EUS-guided FNA specimens from pancreatic masses. Gastrointest Endosc.

[13] Reicher S, Boyar FZ, Albitar M, Sulcova V, Agersborg S, Nga V, Zhou Y, Li G, Venegas R, French SW, Chung DS, Stabile BE, Eysselein VE (2011). Fluorescence in situ hybridization and K-ras analyses improve diagnostic yield of endoscopic ultrasound-guided fine-needle aspiration of solid pancreatic masses. Pancreas.

[14] Tada M, Komatsu Y, Kawabe T, Sasahira N, Isayama H, Toda N, Shiratori Y, Omata M (2002). Quantitative analysis of K-ras gene mutation in pancreatic tissue obtained by endoscopic ultrasonography-guided fine needle aspiration: clinical utility for diagnosis of pancreatic tumor. Am J Gastroenterol.

[15] Maluf-Filho F, Kumar A, Gerhardt R, Kubrusly M, Sakai P, Hondo F, Matuguma SE, Artifon E, Monteiro da Cunha JE, Cesar Machado MC, Ishioka S, Forero E (2007). Kras mutation analysis of fine needle aspirate under EUS guidance facilitates risk stratification of patients with pancreatic mass. J Clin Gastroenterol.

[16] Pellise M, Castells A, Gines A, Sole M, Mora J, Castellvi-Bel S, Rodriguez-Moranta F, Fernandez-Esparrach G, Llach J, Bordas JM, Navarro S, Pique JM (2003). Clinical usefulness of KRAS mutational analysis in the diagnosis of pancreatic adenocarcinoma by means of endosonography-guided fine-needle aspiration biopsy. Aliment Pharmacol Ther.

[17] Ogura T, Yamao K, Hara K, Mizuno N, Hijioka S, Imaoka H, Sawaki A, Niwa Y, Tajika M, Kondo S, Tanaka T, Shimizu Y, Bhatia V (2013). Prognostic value of K-ras mutation status and subtypes in endoscopic ultrasound-guided fine-needle aspiration specimens from patients with unresectable pancreatic cancer. J Gastroenterol.

[18] Kelly KA, Bardeesy N, Anbazhagan R, Gurumurthy S, Berger J, Alencar H, Depinho RA, Mahmood U, Weissleder R (2008). Targeted nanoparticles for imaging incipient pancreatic ductal adenocarcinoma. PLoS Med.

[19] Bausch D, Thomas S, Mino-Kenudson M, Fernandez-del CC, Bauer TW, Williams M, Warshaw AL, Thayer SP, Kelly KA (2011). Plectin-1 as a novel biomarker for pancreatic cancer. Clin Cancer Res.

[20] Stathis A, Moore MJ (2010). Advanced pancreatic carcinoma: current treatment and future challenges. Nat Rev Clin Oncol.

[21] Bausch D, Mino-Kenudson M, Fernandez-Del Castillo C, Warshaw AL, Kelly KA, Thayer SP (2009). Plectin-1 is a biomarker of malignant pancreatic intraductal papillary mucinous neoplasms. J Gastrointest Surg.

[22] Micames C, Jowell PS, White R, Paulson E, Nelson R, Morse M, Hurwitz H, Pappas T, Tyler D, McGrath K (2003). Lower frequency of peritoneal carcinomatosis in patients with pancreatic cancer diagnosed by EUS-guided FNA vs. percutaneous FNA. Gastrointest Endosc.

[23] Spier BJ, Johnson EA, Gopal DV, Frick T, Einstein MM, Byrne S, Koscik RL, Liou JI, Broxmeyer T, Selvaggi SM, Pfau PR (2009). Predictors of malignancy and recommended follow-up in patients with negative endoscopic ultrasound-guided fine-needle aspiration of suspected pancreatic lesions. Can J Gastroenterol.

[24] Winner M, Sethi A, Poneros JM, Stavropoulos SN, Francisco P, Lightdale CJ, Allendorf JD, Stevens PD, Gonda TA (2015). The role of molecular analysis in the diagnosis and surveillance of pancreatic cystic neoplasms. Jop.

[25] Khalid A, Zahid M, Finkelstein SD, LeBlanc JK, Kaushik N, Ahmad N, Brugge WR, Edmundowicz SA, Hawes RH, McGrath KM (2009). Pancreatic cyst fluid DNA analysis in evaluating pancreatic cysts: a report of the PANDA study. Gastrointest Endosc.

[26] Salek C, Benesova L, Zavoral M, Nosek V, Kasperova L, Ryska M, Strnad R, Traboulsi E, Minarik M (2007). Evaluation of clinical relevance of examining K-ras, p16 and p53 mutations along with allelic losses at 9p and 18q in EUS-guided fine needle aspiration samples of patients with chronic pancreatitis and pancreatic cancer. World J Gastroenterol.

[27] Preis M, Gardner TB, Gordon SR, Pipas JM, Mackenzie TA, Klein EE, Longnecker DS, Gutmann EJ, Sempere LF, Korc M (2011). MicroRNA-10b expression correlates with response to neoadjuvant therapy and survival in pancreatic ductal adenocarcinoma. Clin Cancer Res.

[28] Wang Y, Gao J, Li Z, Jin Z, Gong Y, Man X (2007). Diagnostic value of mucins (MUC1, MUC2 and MUC5AC) expression profile in endoscopic ultrasound-guided fine-needle aspiration specimens of the pancreas. Int J Cancer.

[29] Kim ST, Lim do H, Jang KT, Lim T, Lee J, Choi YL, Jang HL, Yi JH, Baek KK, Park SH, Park YS, Lim HY, Kang WK (2011). Impact of KRAS mutations on clinical outcomes in pancreatic cancer patients treated with first-line gemcitabine-based chemotherapy. Mol Cancer Ther.

[30] Whitcombe D, Theaker J, Guy SP, Brown T, Little S (1999). Detection of PCR products using self-probing amplicons and fluorescence. Nat Biotechnol.

[31] Thelwell N, Millington S, Solinas A, Booth J, Brown T (2000). Mode of action and application of Scorpion primers to mutation detection. Nucleic Acids Res.

[32] Newton CR, Graham A, Heptinstall LE, Powell SJ, Summers C, Kalsheker N, Smith JC, Markham AF (1989). Analysis of any point mutation in DNA. The amplification refractory mutation system (ARMS). Nucleic Acids Res.

